# A Combination of Doxycycline and Ribavirin Alleviated Chikungunya Infection

**DOI:** 10.1371/journal.pone.0126360

**Published:** 2015-05-13

**Authors:** Hussin A. Rothan, Hirbod Bahrani, Zulqarnain Mohamed, Teow Chong Teoh, Esaki M. Shankar, Noorsaadah A. Rahman, Rohana Yusof

**Affiliations:** 1 Department of Molecular Medicine, Faculty of Medicine, University of Malaya, 50603 Kuala Lumpur, Malaysia; 2 Institute of biological sciences, Faculty of Science, University of Malaya, 50603 Kuala Lumpur, Malaysia; 3 Department of Medical Microbiology, Faculty of Medicine, University of Malaya, 50603 Kuala Lumpur, Malaysia; 4 Department of Chemistry, Faculty of Science, University of Malaya, 50603 Kuala Lumpur, Malaysia; University of California, San Francisco, UNITED STATES

## Abstract

Lack of vaccine and effective antiviral drugs against chikungunya virus (CHIKV) outbreaks have led to significant impact on health care in the developing world. Here, we evaluated the antiviral effects of tetracycline (TETRA) derivatives and other common antiviral agents against CHIKV. Our results showed that within the TETRA derivatives group, Doxycycline (DOXY) exhibited the highest inhibitory effect against CHIKV replication in Vero cells. On the other hand, in the antiviral group Ribavirin (RIBA) showed higher inhibitory effects against CHIKV replication compared to Aciclovir (ACIC). Interestingly, RIBA inhibitory effects were also higher than all but DOXY within the TETRA derivatives group. Docking studies of DOXY to viral cysteine protease and E2 envelope protein showed non-competitive interaction with docking energy of -6.6±0.1 and -6.4±0.1 kcal/mol respectively. The 50% effective concentration (EC50) of DOXY and RIBA was determined to be 10.95±2.12 μM and 15.51±1.62 μM respectively, while DOXY+RIBA (1:1 combination) showed an EC_50_ of 4.52±1.42 μM. When compared, DOXY showed higher inhibition of viral infectivity and entry than RIBA. In contrast however, RIBA showed higher inhibition against viral replication in target cells compared to DOXY. Assays using mice as animal models revealed that DOXY+RIBA effectively inhibited CHIKV replication and attenuated its infectivity *in vivo*. Further experimental and clinical studies are warranted to investigate their potential application for clinical intervention of CHIKV disease.

## Introduction

Chikungunya virus (CHIKV) infection is a vector-borne disease transmitted to humans by the bite of female *Aedes aegypti* mosquitoes. The clinical significance of CHIKV has been emphasized by the epidemic outbreaks across the Indian sub-continent [1,2]. Since 2004, CHIKV-infected cases recorded across ~40 countries have been estimated to be >1.4–6.5 million [2]. Most of the CHIKV outbreaks were reported from tropical countries particularly across Africa, South-East Asia and the Indian Ocean [3]. *Aedes albopictus* has recently been shown to be a potential vector of CHIKV [4], which likely increases the possibilities of viral distribution across the non-epidemic regions of CHIKV infection such as Europe North America [5]. Very recently in 2014, in Central and South America, 174 cases of locally transmitted disease have been confirmed in El Salvador, Panama, Costa Rica, Venezuela and the Guianas. In addition, four cases of CHIKV infection have been confirmed in southern Florida of United States of America [6].

The lack of anti-viral drugs and vaccines remains a major challenge in the control of CHIKV outbreaks, as current treatment strategies rely on the alleviation of disease symptoms. Common symptoms of CHIKV infection includes high fever (~40°C), headache, back pain, myalgia, vomiting, nausea, conjunctivitis and arthralgia [1]. With the steady increase of CHIKV infection cases in recent years, there is indeed a dire need for newer and effective intervention strategies against this disease. The advent of broad-spectrum antimicrobial agents with minimal side-effects has immensely contributed to the advancement of modern therapeutics in medicine [7,8]. In particular, tetracycline derivatives (TETRA) are commonly used against bacterial infections, although antiviral functions of these antibiotics have not been completely investigated [9,10]. Doxycycline (DOXY), a semi-synthetic tetracycline antibiotic with significant effect against bacterial infections [11–13] works by preventing protein synthesis, and thus expands its usefulness in the clinical treatment of a broad array of bacterial infections [14]. DOXY have been shown to exert inhibitory effects against other microbial infections such as rickettsia, malaria [15] and chlamydia infections [16]. In addition, *in vivo* studies have indicated that treatment with DOXY attenuates acute lung injury in mice infected with virulent influenza H3N2 virus [17]. Interestingly, synergistic effects of DOXY administered together with oseltamivir provide an effective intervention strategy against swine flu infection [18]. Furthermore, *in vitro* studies showed that DOXY also exhibited significant inhibitory effects against antimurine retrovirus [19] and dengue virus replication in infected cell lines [20, 21]. Here, we established that a combination of DOXY and RIBA showed potent antiviral activities by inhibiting entry and replication of CHIKV in Vero cells, and reduced viral infectivity *in vitro* and *in vivo*.

## Methods

### Virus and cells

A clinical isolate of CHIKV (East/Central/South African genotype) was recovered from a patient’s serum and used for virus propagation by a single passage in C6/36 mosquito cells. The viral titre (10^7^ p.f.u./ml) of CHIKV suspension was established by serial dilution on Vero cells using a plaque assay. Vero (African green monkey kidney cells, ATCC CCL-81) were obtained from the American Type Culture Collection (ATCC; Rockville, MD, USA) and cultured in Dulbecco’s modified Eagle’s medium (DMEM) supplemented with 10% fetal bovine serum (FBS) as a growth medium or 2% FBS as a maintenance medium. All of the drugs used in this study were purchased from Sigma (Sigma Aldrich) and dissolved in DMSO to a final concentration of less than 1% of the total volume.

### Cytotoxicity assay

Vero cells (1 × 10^4^ cells per well of a 96-well plate) were treated with increasing concentrations (0, 6.25, 12.5, 25, 50, 100 μM) of the test compound for 72 h. The cytotoxic effect of the test compounds were determined based on an MTT (3-(4,5-dimethylthiazol 2-yl)-2,5-diphenyltetrazolium bromide) assay as previously reported [21]. The percentage of cell viability was calculated as follows: 100 − (Absorbance of treated cells / Absorbance of untreated cells) × 100.

### Evaluation of antiviral activities of tetracycline derivatives

Vero cells were cultured in 24-well plates (1.5 × 10^5^ cells per well) for 24 h at 37°C and 5% CO_2_. Virus culture supernatant was added to the cells at an MOI of 2, followed by incubation for 2 h with gentle shaking every 15 min to achieve optimal virus-to-cell contact. The cells were washed twice with fresh serum-free DMEM after removing the virus culture supernatant. Subsequently, new complete DMEM containing 15 μM of the test compounds was added to the cultures and incubated for 72 h. The viral titre was evaluated using a plaque formation assay.

### Molecular modelling and docking of Doxycycline to CHIKV protease and E2 envelope glycoprotein

The crystal structures for the ligand DOXY (2XRL), the CHIKV protease receptor protein (3TRK) and the E2 envelope glycoprotein (3N41) were obtained from the RCSB Protein Data Bank (PDB) website (http://www.rcsb.org/pdb/home/home.do). The AutoDock Vina [22] molecular docking software was used to investigate the binding of DOXY to CHIKV protease and E2 envelope glycoprotein. The input files for AutoDock Vina were prepared using AutoDock Tools 1.5.6 [23] where a grid box of 30x30x30 Å^3^ was used by the default settings of 10 docked conformations for each run with the Iterated Local Search global optimizer algorithm. The docking simulation run was repeated three times and all data were presented as mean ± standard error. Lowest docked energy infers the best binding of ligand to receptor and the docked conformation images were produced by PyMOL 1.3 molecular modelling software, as well as for the hydrogen bond analysis.

### Viral inactivation assay

We employed a virus inactivation assay to assess the ability of the test compounds to inactivate CHIKV and prevent subsequent infection,. Vero cells were seeded in 24-well microplates (1.5 x 10^5^ cells/well) for 24 hrs. The test compounds (15 μM of DOXY or RIBA and a 15 μM mixture (1:1) of DOXY+RIBA) were mixed with the virus culture supernatant (MOI of 2) and incubated at 37°C for 1 h and then inoculated onto the Vero cells with gentle shaking every 15 min for 2 h. The virus and test compound mix was then removed, and the cells were washed twice with fresh serum-free DMEM to remove any residual virus. New complete DMEM was later added, and the cultures were incubated for 72 h at 37°C supplemented with 5% CO_2_. The viral titre of the CHIKV suspension was determined through plaque formation assays.

### Viral attachment assay

This assay was performed to determine the ability of the test compounds to inhibit virus entry into host cells. Vero cells were grown in 24-well microplates (1.5 x 10^5^ cells/well) for 24 hrs. Cell culture media were removed and the cells were then washed three times with PBS. New media containing virus and test compound mix (concentrations as previously described) were separately added and the cells were incubated for 1 hr at 4°C. The media were later removed and the cells were washed extensively with cold PBS to remove unadsorbed virus. Cells were incubated for 72 h and the viral titre was quantified by plaque formation assays.

### Post-infection assay

To investigate the inhibitory effect of the test compounds against CHIKV post-infection, Vero cells were seeded in a 24-well tissue culture plate (1.5 × 10^5^ cells/well), incubated for 24 h under optimal conditions and infected with CHIKV (MOI of 2). Post-infection treatment was performed after inoculating Vero cells with CHIKV for 2 h at 37°C, and subsequently complete DMEM media containing the test compounds (15 μM of DOXY or RIBA and a 15 μM mixture (1:1) of DOXY+RIBA) were added. The cultures were then incubated for 72 h at 37°C and 5% CO_2_; three wells of infected cells for each experiment were maintained without test compounds as controls and the viral titre was quantified by plaque formation assays. Cell viability was measured after infection and treatment by MTT assay.

### Virus quantification by plaque formation assay

A 10-fold serial dilution of culture supernatant of CHIKV- infected cells was added to fresh Vero cells grown in 24-well plates (1.5 × 10^5^ cells) and incubated for 1 h at 37°C. The cells were overlaid with DMEM containing 1.1% methylcellulose. Viral plaques were stained with crystal violet dye after 5-day incubation. Virus titres were calculated according to the following formula: Titre (p.f.u./ml) = number of plaques × volume of the diluted virus added to the well × dilution factor of the virus used to infect the well in which the plaques were enumerated.

### ELISA-like cell-based assay

A dose response assay was performed to determine the effect of DOXY, RIBA and DOXY+RIBA on CHIKV replication in Vero cells. The concentrations of test compounds DOXY and RIBA were 0, 5, 10, 15 and 20 μM while the final concentrations of DOXY+RIBA were 0, 5, 10, 25, 50 and 100 μM. The assay was initiated by seeding Vero cells at 1 ×10^4^ cells/well (carried out in triplicate) under optimal conditions (37°C, 5% CO_2_ in a humidified incubator) in 96-well plates. Virus was mixed with test compounds (MOI of 2) and inoculated to the cells followed by incubation at 37°C for 2 h with gentle shaking every 15 min. At the end of incubation, cells were washed three times with PBS and incubated for 72 h. Culture media was then removed and cells were washed again with PBS before fixing with ice-cold methanol for 15 min at -20°C. After these washing steps, cells were then incubated with blocking buffer containing bovine serum albumin for 1 h at room temperature. CHIKV antibody (Abcam, UK, Cat. no. ab126796) was later added, and cells were left to incubate overnight at 4°C. Following overnight incubation, cells were washed three times with PBS and incubated for 30 min with anti-mouse IgG conjugated with alkaline phosphatase. The cells were then washed again with PBS and the absorbance between 490–650 nm was measured using an ELISA reader. EC50 was calculated using non-linear regression fitting (GraphPad Prism version 5.01).

A similar experimental procedure was carried out for different time points (4, 8, 24, 48 and 72 h) following treatment of infected cells with 15 μM of DOXY or RIBA, or 15 μM of DOXY+RIBA (1:1). The results of ELISA experiments were recorded as mean of the 6-plicate experiments.

### Antiviral and toxicity testing *in vivo*

Animal experiments were carried out in accordance with the University of Malaya guidelines on the Care and Use of Laboratory Animals, and research protocols applied in the investigation had been approved by the Animal Ethics Committee of the University of Malaya. Adult ICR mice were used in acute toxicity and viral infection experiments. Acute toxicity assay was performed by administering the test compounds individually or in combination intraperitoneally to three groups of animals (n = 6 each group). The first two groups of animals were given low dose (5 mg/kg) and high dose (50 mg/kg) respectively, while the third group was kept without treatment The range of the doses was selected based on previous *in vivo* studies on RIBA [24, 25] and DOXY [26]. The animals were observed for 24 h for signs of toxicity, and after 14 days post-treatment the animals were sacrificed for histological examinations. Viral infection experiments was performed using four groups of animals (n = 8 each). The four groups included positive control (animals were intraperitoneally inoculated with 1 × 10^6^ plaque-forming units (PFU) of the purified CHIKV as described previously [27], RIBA-treated group (infected-animals treated with 15 mg/kg of RIBA), DOXY+RIBA-treated group (infected-animals treated with 15 mg/kg of RIBA and 10 mg/kg of DOXY) and the fourth group was administrated with PBS as a mock-administrated group. Single dose of the test compounds were administrated intraperitonally after 6 h post-infection. Mice were observed for 7 days post-infection and sacrificed for viral quantification and histopathology analysis. Spleen and liver were collected, weighed and fixed in 4% formaldehyde in PBS, and embedded in paraffin using a Leica ASP300 S tissue processor and a Leica EG1160 embedding station (Leica). Sections (4 μm) were stained with hematoxylin–eosin (H&E).

### Statistical analysis

All of the assays were performed in triplicate, and the statistical analyses were performed using GraphPad Prism version 5.01 (GraphPad Software, San Diego, CA). P values of <0.05 were considered significant.

## Results

### Determination of potential cytotoxic effects of antimicrobial agents on Vero cells

We determined the potential cytotoxic effects of antimicrobial agents tested in the current investigation on Vero cells. TETRA derivatives were evaluated for their antiviral properties against CHIKV, and therefore the cytotoxic effects of these compounds were first evaluated. The maximal non-toxic dose (MNTD value) was determined in this study to be approximately 25 μM that showed 80% cell viability. Therefore, all subsequent *in vitro* cell culture experiments were carried out using doses less than 25 μM for all compounds under study (S1 Fig).

### DOXY and RIBA showed increased anti-CHIKV activity by plaque formation assay

**Fig 1:**
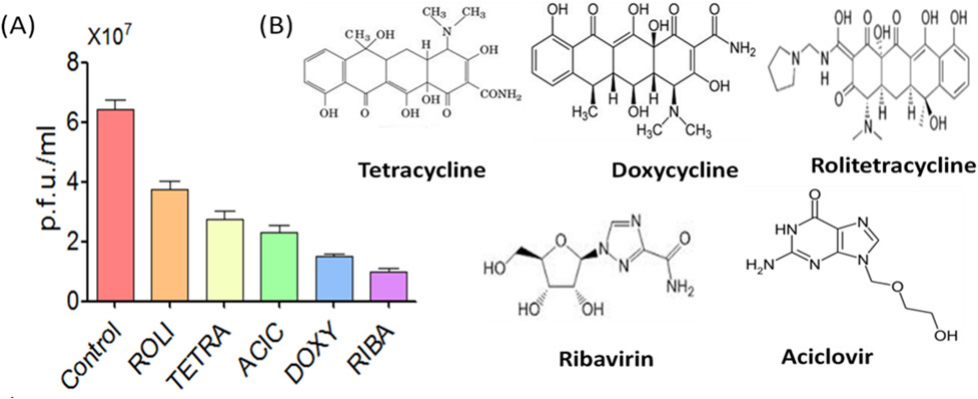
The inhibitory effect of antimicrobial agents against CHIKV replication in Vero cells. **(A)** Viral load was significantly reduced after the treatment with TETRA derivatives and the antiviral drugs compared with the untreated CHIKV-infected cells. DOXY showed highest inhibitory effect against CHIKV replication in Vero cells compared to the control, ROLI and TETRA. RIBA showed higher inhibitory effect (p<0.01) against CHIKV compared to ACIC and other TETRA derivatives except DOXY. **(B)** The chemical structure of the medical compounds (One way ANOVA, P<0.01).

The antiviral activity of TETRA derivatives (TETRA, DOXY and ROLI) and the antiviral drugs (ACIC and RIBA) against CHIKV was investigated by plaque formation assay (S2 Fig). Our results showed that the CHIKV load expressed as plaque forming units per ml was significantly reduced following treatment with TETRA derivatives in addition to the antiviral drugs (One Way ANOVA, P<0.01) compared with untreated cells (Fig 1A and 1B). Interestingly, DOXY showed highest inhibitory effect against CHIKV replication in Vero cells (1.50 × 10^7^ p.f.u./ml) compared to the control (6.43 × 10^7^ p.f.u./ml), ROLI (3.76 × 10^7^ p.f.u./ml) and TETRA (2.76 × 10^7^ p.f.u./ml). In addition, RIBA showed higher inhibitory effect (P<0.01) against CHIKV (0.96 × 10^7^ p.f.u./ml) compared to ACIC (2.30 × 10^7^ p.f.u./ml) and other TETRA derivatives except DOXY. Based on these results, we concluded that two antimicrobial agents i.e DOXY and RIBA showed high virus inhibition potential against CHIKV and were therefore selected for further anti-CHIKV investigations.

### Docking studies of DOXY to CHIKV cysteine protease and E2 glycoprotein

**Fig 2:**
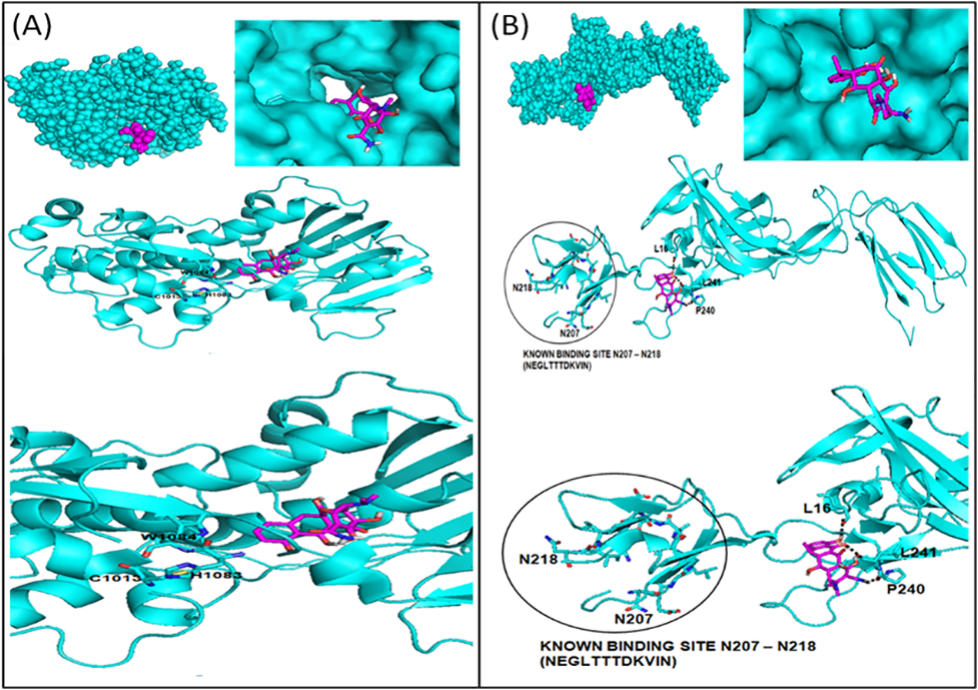
Docking studies of DOXY to CHIKV cysteine protease and E2 glycoprotein. The input files for AutoDock Vina were prepared using AutoDock Tools 1.5.6 where a grid box of 30x30x30 Å^3^ was used by the default settings of 10 docked conformations for each run with the Iterated Local Search global optimizer algorithm. **(A)** DOXY and viral protease binding is due to non-polar and van der Waal interactions. The predicted binding of DOXY to protease was not at the binding site indicating to possible non-competitive inhibition of DOXY against CHIKV protease. **(B)** Binding of DOXY to viral E2 glycoprotein involved the amino acids L16, P240 and L241 in addition to known E2 binding site to cell receptors (circled).

DOXY showed best binding with the CHIKV cysteine protease (docking energy, -6.6±0.1 kcal/mol), and similar binding was observed with CHIKV E2 glycoprotein (docking energy,-6.4±0.1 kcal/mol) for 10 docked conformations. Since there is no hydrogen bond between DOXY and the viral protease, the interaction is theoretically due to non-polar and van der Waal interactions (Fig 2A). In addition, the predicted binding of DOXY to E2 glycoprotein did not occur at the predicted binding site that involves the residues N207 to N218. DOXY binding with E2 glycoprotein was through the hydrogen bonds that were initiated with L16, P240 and L241 residues of E2 glycoprotein on the basis of most negative docked energy by AutoDock Vina (Fig 2B).

### DOXY+RIBA led to considerable reduction of CHIKV particles in Vero cells

**Fig 3:**
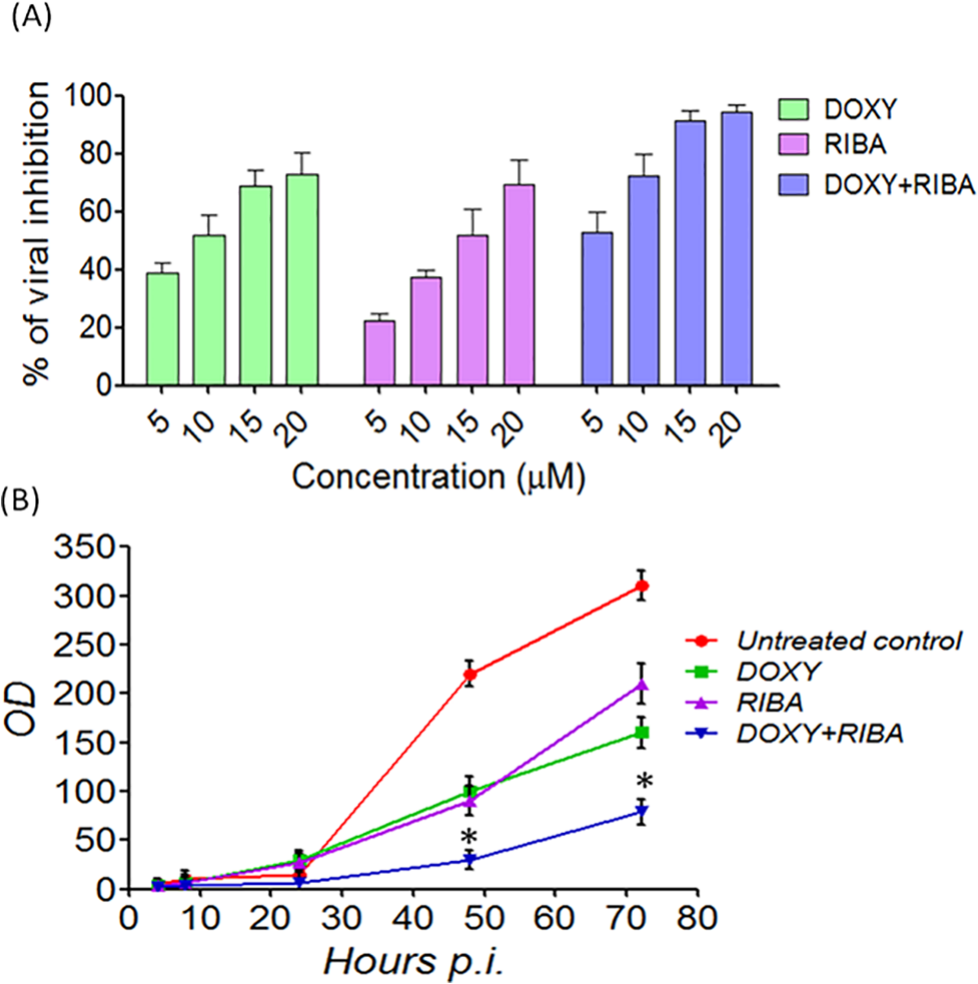
Inhibition of CHIKV after the treatment with DOXY, RIBA and DOXY+RIBA. **(A)** ELISA-like cell-based assay was used to evaluate the antiviral activity of the drugs. Viral antigen was detected by CHIKV antibody and anti-mouse IgG conjugated with alkaline phosphatase. The 50% effective concentration (EC_50_) of the DOXY was calculated to be 10.95±2.12 μM and the RIBA drug was 15.51±1.62 μM, while DOXY+RIBA (1:1) showed an EC_50_ of 4.52±1.42 μM. **(B)** Levels of viral proteins after the treatment with drugs were measured by ELISA-like cell-based assay of triplicates at 4, 8, 24, 48 and 72 h post infection. The OD values were measured by an ELISA reader at 490–650 nm. The result showed considerable improvement on performance when the test compound were administered together compared to individual treatment (Two way ANOVA, *P<0.05).

Given that DOXY and RIBA showed increased inhibition potentials against CHIKV in our investigations, we next characterised the antiviral effectiveness of DOXY, RIBA and DOXY+RIBA, against CHIKV using a ELISA-like cell-based assay and fluorescence microscopy following immunostaining. Our results showed that the test compounds inhibited CHIKV replication in Vero cells in a dose-dependent manner (Fig 3A). The 50% effective concentration (EC_50_) for DOXY was estimated to be 15.51±1.62 μM and for RIBA it was 10.95±2.12 μM, while DOXY+RIBA (1:1) showed an EC_50_ of 4.52±1.42 μM. Interestingly, DOXY+RIBA showed 95% viral inhibition at 15 μM, even though at same concentration DOXY and RIBA showed only 51% and 68% of viral inhibition respectively (Fig 3A). Infectious particles of CHIKV were reduced after the treatment with DOXY, RIBA and DOXY+RIBA in a time dependent manner with considerable improvement on performance when the test compound were administered together (Fig 3B). Together, we concluded that DOXY+RIBA led to considerable improvement in the reduction of CHIKV particles in Vero cells.

### DOXY+RIBA inhibited viral entry of Vero cells

**Fig 4:**
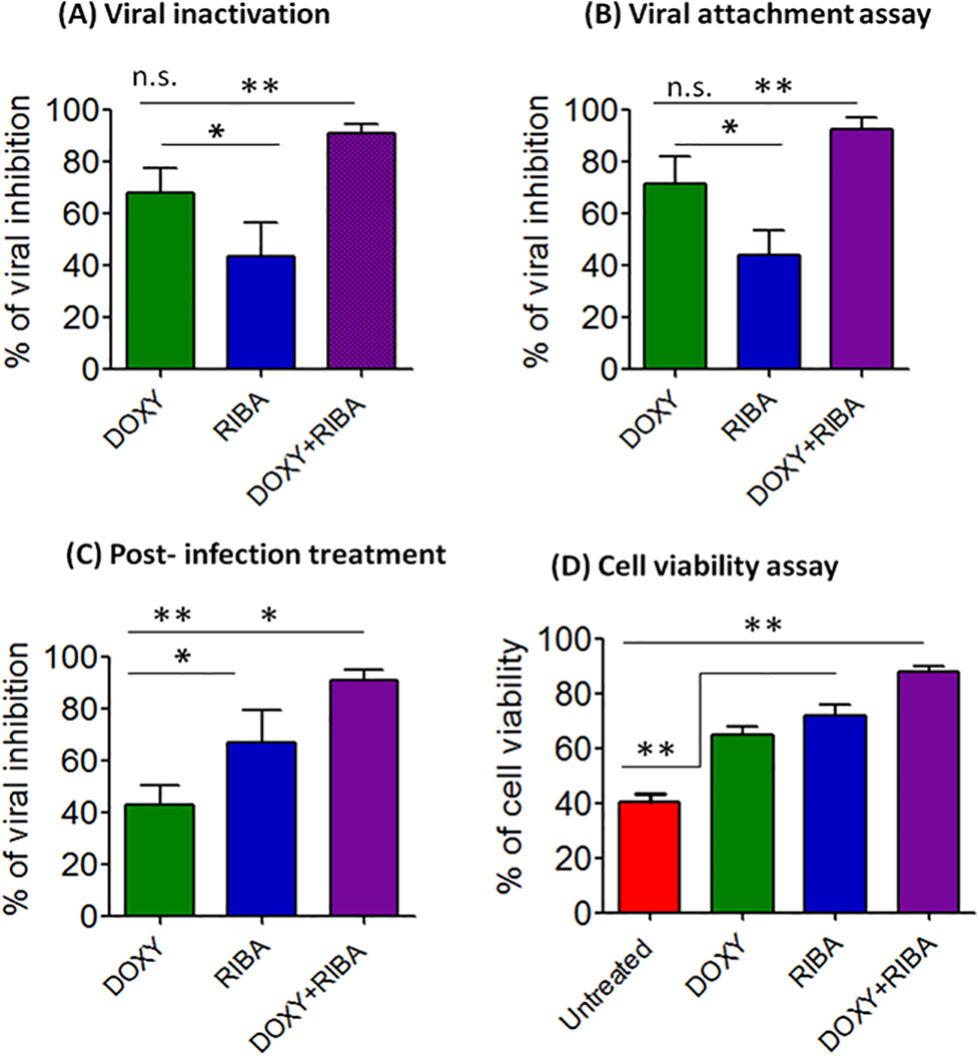
Mode of inhibition of DOXY and its combination with RIBA against CHIKV. **(A)** Viral inactivation assay showed significant viral inhibition after the treatment with DOXY compared with RIBA; while DOXY+RIBA showed the highest inhibition percentage. **(B)** Viral attachment assay showed that DOXY significantly inhibited virus attachment to target cells compared to RIBA and the combination of DOXY+RIBA showed the highest inhibitory effect. **(C)** Post-infection treatment showed that DOXY inhibitory effect against CHIKV was lower than the inhibitory effect of RIBA. The combination treatment significantly inhibited virus replication compared with the individual treatments. **(D)** Cell viability of CHIKV-infected cells after treatment with DOXY, RIBA and a DOXY+RIBA compared with untreated cells (One Way ANOVA, *P<0.05, **P<0.01).

We investigated whether DOXY+RIBA act by inhibiting viral entry of Vero cells. First, we incubated CHIKV with the test compound and then inoculated the cells to examine whether the compounds can inactivate CHIKV and inhibit subsequent events of viral entry (Fig 4A). Treatment of the infected cells with DOXY+RIBA showed the highest inhibitory effect (91%) compared with individual compounds. The difference between DOXY+RIBA and DOXY alone was statistically insignificant (One Way ANOVA, P>0.05); while it was highly significant (One Way ANOVA, P< 0.01) compared to RIBA. In addition, DOXY showed approximately 68% of viral inhibition compared to 45% of RIBA (P<0.05). Similarly, the results of viral attachment assay showed that DOXY+RIBA exhibited the highest inhibition effects against viral infectivity (92%) as represented in Fig 4B. The results showed potential inhibition of DOXY (71%) compared with RIBA (44%) against CHIKV (One Way ANOVA, P<0.05).

### Presence of DOXY+RIBA in the culture media significantly reduced CHIKV replication post-infection

We then incubated Vero cells with CHIKV at 37°C for 2 h and subsequently treated the cells with the test compounds to determine their inhibitory effects against viral replication post-infection. The inhibitory effect of DOXY against CHIKV post-infection was lower (43%) than the inhibitory effect showed by RIBA (67%). The presence of the two drugs in the culture media of the infected cells was significantly more effective (One Way ANOVA, P<0.01) in reducing viral replication (91%) compared to individual treatments with DOXY or RIBA (Fig 4C). The MTT assay showed that the viability of CHIKV-infected cells was significantly (One Way ANOVA, P<0.01) improved after treatment with DOXY (65%), RIBA (71.6%) and DOXY+RIBA (88.2%) compared with untreated cells (40.3%) as presented in Fig 4D.

### Morphological and histological changes in the liver and spleen of CHIKV-infected mice after the treatment with DOXY+RIBA

Since the *in vitro* results showed the DOXY+RIBA treatment led to improved inhibition in CHIKV replication, we next set out to examine if DOXY+RIBA treatment have an effect on reducing CHIKV viral load and infectivity *in vivo* compared to the general antiviral drug RIBA. The experiment of acute toxicity was performed by administering the animals with low (5 mg/kg) and high dose (50 mg/kg) of the drugs, individually or in combination, intraperitoneally. The test compounds at these doses showed no signs of toxicity throughout the 24 h of observation. Histological examinations of liver, spleen and kidney were similar with untreated control group after up to 14 days post-treatment (data not shown).

**Fig 5:**
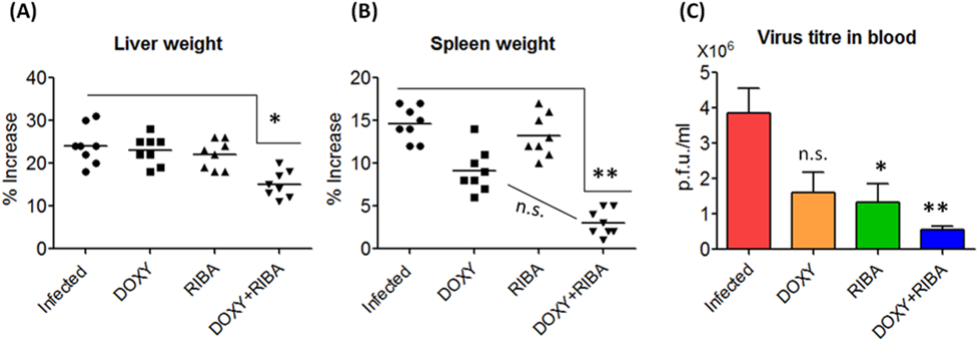
CHIKV-infected mice showed liver and spleen hypertrophy, but reduction in size was evident after treatment. **(A)** Treatment with DOXY+RIBA led to significant reduction in liver weight (p<0.05) compared with infected group. **(B)** Treatment with DOXY+RIBA showed considerable reduction in spleen weight (p<0.01), as evidenced by Kruskal-Wallis test followed by Dunn’s post-test. **(C)** Viral titer in blood of infected mice was quantified by plaque formation assay (limit of detection was 10 pfu) after 7 days post infection. Although RIBA significantly (One Way ANOVA, P<0.05) reduced viral titer, DOXY+RIBA showed a more prominent reduction compared to control (One Way ANOVA, P<0.01).

To observe the effects of drug treatment on cell morphology and histology, CHIKV-infected mice were first grouped into DOXY-, RIBA- and DOXY+RIBA- treated animals besides infected animals without treatment and normal animals as controls. After 7 days post-infection, CHIKV-infected mice usually show liver and spleen hypertrophy (Image A in S3 Fig). The liver of non-treated CHIKV-infected mice were also larger and irregular in shape compared to normal controls. We noticed that treatment with DOXY+RIBA resulted in considerable enhancement in liver size and shape, although these effects were not observed when mice were treated with DOXY or RIBA alone (Image B in S3 Fig). The spleen of [non-treated] CHIKV-infected mice also increased in size compared to normal mice. However, it was noted that DOXY+RIBA treatment markedly reduced the size of the spleen. Again, treatment with either DOXY or RIBA alone showed insignificant effect on spleen size (Image C in S3 Fig). To support these observation, liver and spleen weight was also measured as viral infection is expected to cause considerable weight increase. A similar trend of results were obtained, where treatment with DOXY+RIBA led to a significant reduction in liver (p<0.05) and spleen (p<0.01) weight compared to individual treatment with DOXY or RIBA (Fig 5A and 5B). Quantification of viral titer in the blood of infected mice by plaque formation assay also showed that treatment with DOXY+RIBA exhibited a significantly higher reduction compared to control (ONE WAY ANOVA, P<0.01). We also noted that although treatment with RIBA significantly (ONE WAY ANOVA, P<0.05) reduced viral titer, treatment with DOXY alone showed insignificant reduction in viral load (Fig 5C).

**Fig 6:**
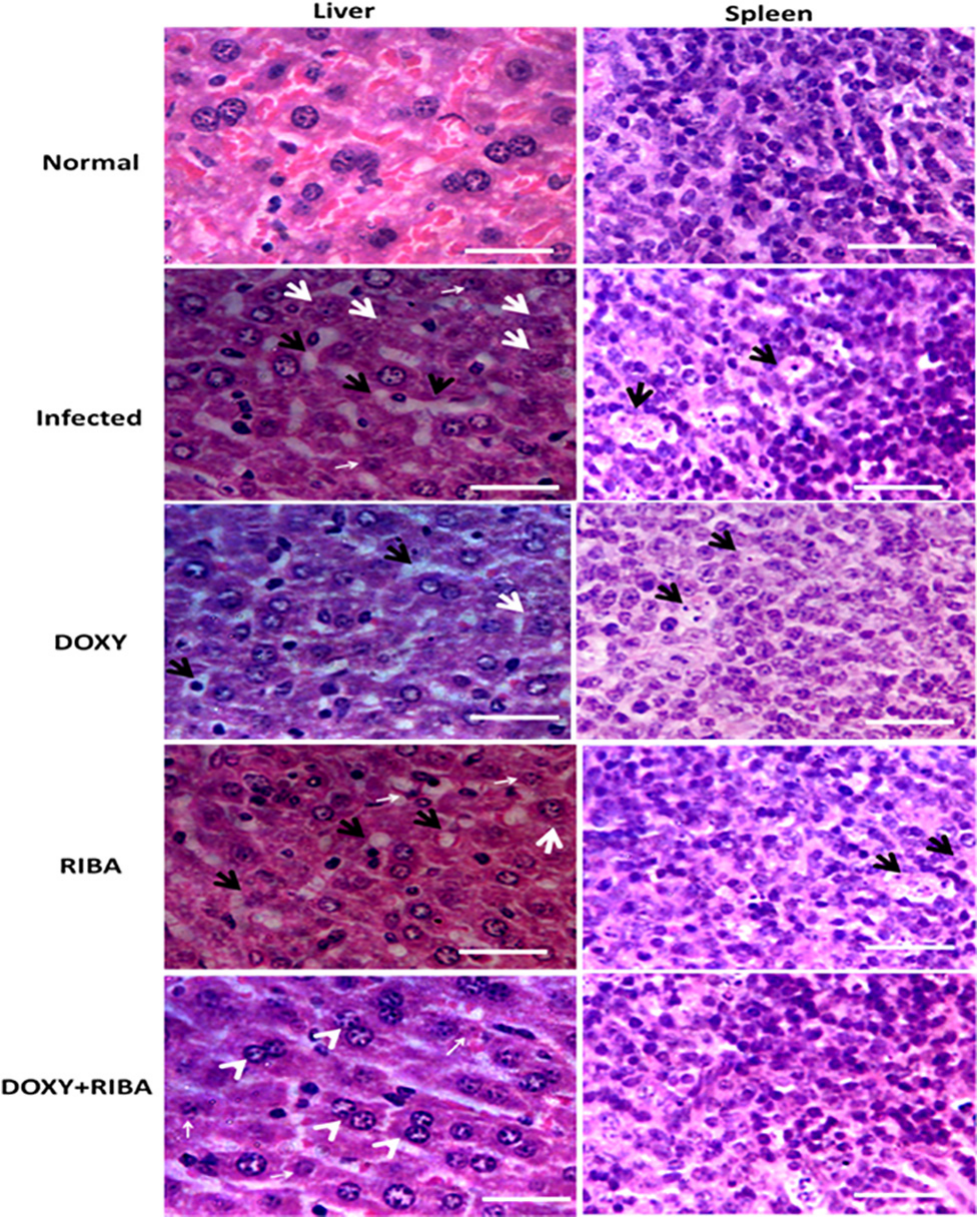
Histopathology of the liver and spleen of CHIKV-infected mice after the treatment with DOXY-RIBA. The liver of infected mice showed accumulated infiltrated fluid due to inflammation (black arrows), many swollen hepatocyte (white arrows) and few shrinking apoptotic cells (small arrows). DOXY- or RIBA- treated mice showed reduction in abnormal cells and inflammation. DOXY+RIBA showed lesser shrinking apoptotic cells (small arrows) while other infection features disappeared. The existence of binucleate hepatocytes indicated accelerated liver cell renewal (arrowheads). The spleen of infected mice showed shrinking apoptotic cells and accumulated infiltrated fluid due to inflammation (black arrows), and this symptoms were reduced due to treatment with DOXY+RIBA (scale bars 50 μM).

The liver of infected mice showed accumulated infiltrated fluid due to inflammation, with high presence of swollen hepatocyte and few shrinking apoptotic cells (Fig 6). DOXY or RIBA-treated mice showed reduction in abnormal cells and inflammation. Treatment with DOXY+RIBA showed lesser amounts of shrinking apoptotic cells while other infection features disappeared. In addition, we note the existence of binucleate hepatocytes suggesting accelerated liver cell renewal. Similarly, the spleen of infected mice showed shrinking apoptotic cells and accumulated infiltrated fluid due to inflammation, and this symptoms were reduced when treated with DOXY+RIBA (Fig 6).

## Discussion

Current treatment strategies against CHIKV infection are aimed at alleviating the symptoms associated with CHIKV disease, and this is largely due to unavailability of effective anti-CHIKV drugs or vaccines for human use. Furthermore, treatment with common antiviral drugs like RIBA or ACIC is almost always ineffective due to unsuitable mechanism of action possessed by these drugs. For instance, it has been recently reported that Hepatitis C virus (HCV) replication in persistently infected cultures induces an autophagy response that impairs RIBA uptake by preventing the expression of equilibrative nucleoside transporter 1 [28]. To increase the effectiveness of this drug, RIBA was administered in combination with other antiviral agents, and results have suggested significant improvement in their antiviral activities against HCV infection, such as pegylated interferon-alpha2a [29, 30], peginterferon alfa-2a and telaprevir [31] and alpha-cyclodextrin [32]. Furthermore, the combination of RIBA with thiosemicarbazone derivative have resulted in considerable success against Japanese Encephalitis Virus [33], whereas a combination of RIBA with cidofovir showed high activity against human adenovirus [34]. Because most of these compounds are relatively expensive, an alternative combination of RIBA with other common antibiotics that showed significant antiviral activities such as DOXY is an attractive alternative. Previous studies have indeed shown that DOXY possessed significant antiviral activity against influenza virus [18], retrovirus [19] dengue virus [20, 21]. To that end, the current study was designed to evaluate the inhibitory effects of TETRA derivatives and certain common antiviral drugs (RIBA and ACIC) against CHIKV infection *in vitro* and *in vivo*.

Our observations showed that viral yield was significantly reduced in CHIKV-infected cells after treatment with TETRA derivatives and other antiviral drugs. Of particular note, outstanding inhibitory effects against CHIKV were observed following treatment with DOXY individually or in combination with RIBA. In the present investigation, we determined the inhibitory effects of DOXY against CHIKV as well as its possible effects with the virus life cycle in Vero cells. In parallel with our previous reports [21, 35] the current study showed that TETRA derivatives exhibited similar cytotoxicity effects where the CC_50_ values were more than 100 μM. On the other hand, both RIBA and ACIC showed lower cytotoxic effects against Vero cells. TETRA derivatives at the doses with more than 80% cell viability showed various levels of the inhibitory effects against CHIKV. Of these, DOXY inhibited CHIKV at the level similar to RIBA. Previous studies have also reported similar antiviral functions of DOXY against various viruses [19–21]. For instance, the antiviral activity of DOXY has been reported against retroviruses where a significant reduction in retrovirus titre was observed after incubation of infected cells with DOXY [19]. Our previous work showed that DOXY inhibits dengue serine protease (NS2B-NS3 protease) and virus entry into the target cells [21]. In this study, when the virus and drug were exposed concurrently on Vero cells, we found that DOXY inhibited CHIKV at EC_50_ of 10.9 μM, which is lower than RIBA (15.5 μM). RIBA has been shown to possess broad spectrum antiviral effects that act by inhibiting viral replication [36]. Based on this fact we postulated that the inhibition effect of DOXY is directed more towards viral entry rather than viral replication. The combination of DOXY and RIBA showed the highest inhibitory effect (EC_50_, 4.5 μM). This could be due to the fact that the combination of these two drugs led to the targeting of virus life cycle at both the entry stage (by DOXY) and at the RNA replication stage (by RIBA). Therefore, we performed additional experiments and assays to delineate the antiviral activity of DOXY alone or in combination with RIBA in the aspects of viral infectivity, entry and replication.

Our computational studies showed the predicted binding of DOXY to CHIKV cysteine protease occur at the active site that is important to catalyse viral polyprotein and then viral replication. The active site of CHIKV cysteine protease was reported to involve W1084, C1013 and H1083 residues [37]. Therefore, DOXY may be considered as a non-competitive inhibitor against CHIKV protease. Furthermore, the computationally predicted binding site of CHIKV E2 glycoprotein involves residues N218 to N207 [38]. Although the binding of DOXY with E2 glycoprotein was not at the expected binding site, DOXY showed binding affinity with L16, L241 and P240 through three hydrogen bonds. This binding may impair the important conformational changes of E2 binding site to bind with cell membrane receptors that eventually facilitate virus entry. Previous computational docking studies with dengue virus have indeed shown that DOXY inhibits virus plaque formation by disrupting the conformational changes in the viral envelope that are necessary for virus entry [20]. To verify these findings, further analysis was carried out *in vitro* to prove that DOXY can inactivate CHIKV and prevent virus entry.

DOXY showed higher potential in reducing viral entry into target cells compared with RIBA. CHIKV was inactivated after 1 h incubation with DOXY, indicating that the drug may have an ability to bind with virus envelop. Similar potential was observed when the virus was incubated with DOXY and cells at 4°C showing that the drug inhibited viral entry into the target cells. Taken together, these results are consistent with our computational data that showed binding affinity between DOXY and CHIKV envelop that may impair important conformational changes of E2 protein during virus entry. Such activity demonstrates the ability of DOXY in inhibiting virus propagation at the cell entry stage. The literature showed broad-spectrum antiviral activity of DOXY [17–20]. Thus, the other mechanism of inhibition that compromise the possible binding of DOXY with specific cell membrane components needs to be considered. Interestingly, post-infection treatment showed low potential effects of DOXY against viral replication in the target cells compared to RIBA, confirming that the main inhibitory effect of DOXY at the entry stage of virus infection.

Our *in vivo* study showed that treatment with DOXY+RIBA led to significant reduction in virus titre in the blood of ICR mice at 7 days post-infection. In this study we used high dose (10^6^ PFU/mice) of CHIKV to examine the ability of the drugs to combat viral infection. This might result in longer persistence of the virus in the serum of ICR mice. It has been illustrated that mice age, strain and dose of inoculation have an effect on virus persistence post-infection. For example, CHIKV genomic RNA persisted in muscle tissues at least 3 weeks post- infection in 14-day-old C57BL/6J mice [39]; while viral RNA was eliminated after 5–6 days in 6- to 10- weeks mice of the same strain [40]. In addition, C57BL/6 mice showed early clearance of HSV-1 in the brain stem when compared with ICR mice [41]. It has been reported that high inoculation dose has a significant correlation the period and level of the viremia in monkey model [42] and in C57BL/6J mice (3- to 5- weeks old up to 7- to 10- days post- infection) [27]. The results of this study are consistent with previous studies that showed CHIKV persistence in the serum for 7- to 10- days post-infection. [27, 39].

The results of histopathology study indicated that viral infection causes the accumulation of infiltrated fluid due to inflammation, with high presence of swollen hepatocyte and few shrinking apoptotic cells in the liver and spleen. Treatment with DOXY+RIBA led to considerable reduction in pathological signs compared with individual treatment with DOXY or RIBA. Similar studies had shown that combining DOXY with oseltamivir, an antiviral drug, provides an effective treatment for swine origin influenza infected patient [18]. In addition, combining DOXY with monocaprin (1-monoglyceride of capric acid, an anti-herpes- virus drug) provides an effective treatment for herpes labialis, significantly reducing time to healing and pain compared with monocaprin alone [43]. This observation could be attributed to the antiviral activity of DOXY in addition to its feature as an anti-inflammatory drug. It has been reported that DOXY is an anti-inflammatory agent based on its potential inhibition of matrix metalloproteinases such as collagenase, gelatinase and stromelysin, which are part of the inflammatory response and contribute to the breakdown of body tissues [43, 44]. Therefore, in this study, the *in vivo* investigation showed a convincing outcome of the combined treatment (DOXY+RIBA) compared with RIBA alone, to mice infected with CHIKV. We postulate the anti-CHIKV activity of DOXY+RIBA would potentially inhibit CHIKV and assist in attenuating viral clinical symptoms due to the anti-inflammatory effects of DOXY.

In conclusion, we convincingly showed that DOXY exhibited significant anti-CHIKV activity that acts by impairing viral entry of Vero cells. DOXY+RIBA reduced viral infectivity and replication in infected cells. Further experimental and clinical studies should be carried out to investigate their potential utilization for the attenuation of the clinical symptoms pathognomonic to CHIKV disease.

## Supporting Information

S1 Fig Cell viability assay. Vero cells were treated with increased concentrations (0, 6.25, 12.5, 25, 50, 100 μM) of the drugs for 72 h in 6-plicates for each drug. The maximal non-toxic dose was determined to be approximately 25 μM, that showed 80% cell viability and above. Therefore, all subsequent *in vitro* cell culture experiments were carried out using doses less than 25 μM for all tested compounds.
(TIF)

S2 Fig Plaque assay image showing the reduction in plaque formation due to treatment with the drugs. Viral load expressed as plaque forming units per ml (p.f.u./ml) was significantly reduced after the treatment with TETRA derivatives and the antiviral drugs compared with the untreated CHIKV-infected cells.
(TIF)

S3 Fig Changes in the liver and spleen weight of CHIKV-infected mice after the treatment with RIBA and DOXY. **(A)** Mice were infected with CHIKV and treated with RIBA alone or in combination with DOXY. After 7 days post-infection, CHIKV-infected mice showed liver and spleen hypertrophy **(B)** Liver of CHIKV-infected mice was larger and irregular compared to normal. DOXY+RIBA showed considerable enhancement in liver size and shape but not RIBA or DOXY alone. **(C)** Spleen of CHIKV-infected mice increased in size compared to normal. DOXY+RIBA treatment successfully reduced the size of spleen while treatment with either RIBA or DOXY individually showed insignificant effect on spleen size.
(TIF)
