# Supplementary figures and images for: A Combination of Doxycycline and Ribavirin Alleviated Chikungunya Infection
Source: PLoS One. 2015 May 13;10(5):e0126360. doi: 10.1371/journal.pone.0126360 (PMC4430285; doi:10.1371/journal.pone.0126360)

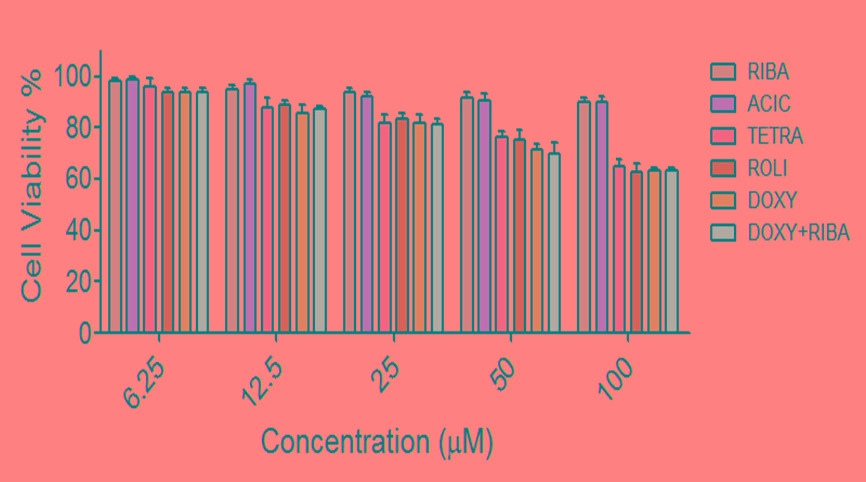

Supplement: S1 Fig — Vero cells were treated with increased concentrations (0, 6.25, 12.5, 25, 50, 100 μM) of the drugs for 72 h in 6-plicates for each drug. The maximal non-toxic dose was determined to be approximately 25 μM, that showed 80% cell viability and above. Therefore, all subsequent in vitro cell culture experiments were carried out using doses less than 25 μM for all tested compounds. (TIF) [file pone.0126360.s001.tif]

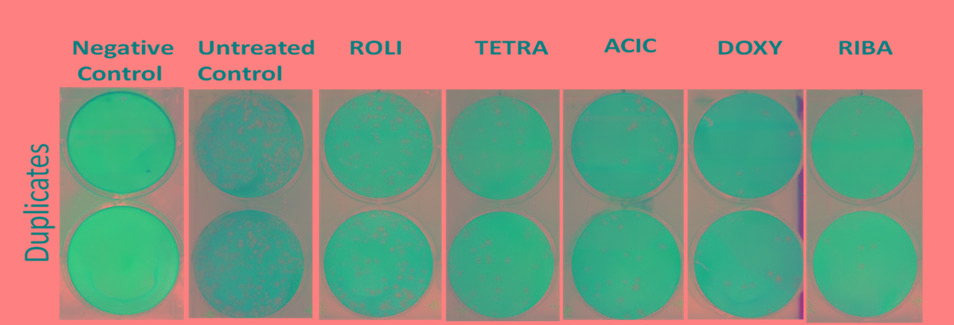

Supplement: S2 Fig — Viral load expressed as plaque forming units per ml (p.f.u./ml) was significantly reduced after the treatment with TETRA derivatives and the antiviral drugs compared with the untreated CHIKV-infected cells. (TIF) [file pone.0126360.s002.tif]

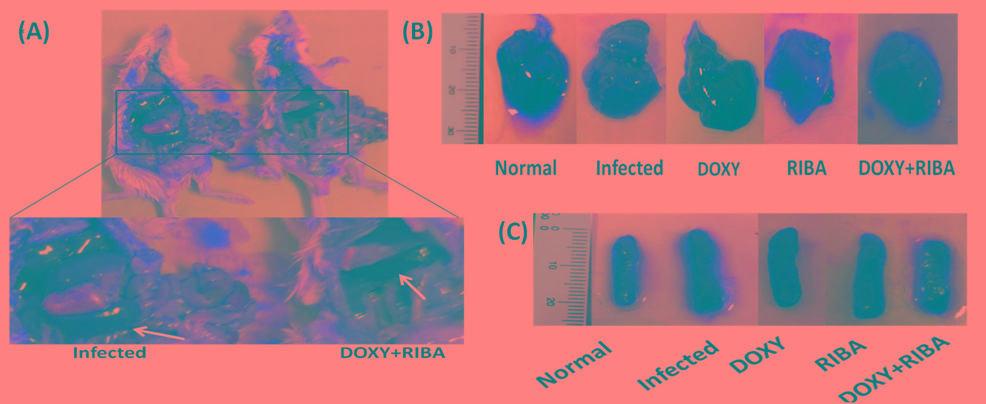

Supplement: S3 Fig — (A) Mice were infected with CHIKV and treated with RIBA alone or in combination with DOXY. After 7 days post-infection, CHIKV-infected mice showed liver and spleen hypertrophy (B) Liver of CHIKV-infected mice was larger and irregular compared to normal. DOXY+RIBA showed considerable enhancement in liver size and shape but not RIBA or DOXY alone. (C) Spleen of CHIKV-infected mice increased in size compared to normal. DOXY+RIBA treatment successfully reduced the size of spleen while treatment with either RIBA or DOXY individually showed insignificant effect on spleen size. (TIF) [file pone.0126360.s003.tif]
